# Chronic rhinitis in HTLV-1 carriers: a histopathologic study

**DOI:** 10.1590/S1808-86942012000200007

**Published:** 2015-10-20

**Authors:** Fernando P. Gaspar Sobrinho, Adelmir Souza-Machado, Álvaro A. Cruz, Hélio A. Lessa, Eduardo A. Ramos

**Affiliations:** a PhD in Medicine (Assistng Physician in the ENT Service of the Professor Edgar Santos University Hospital at the Federal University of Bahia); b PhD in Medicine (Professor in the Department of Biomorphology at the Health Sciences Insttute of the Federal University of Bahia); c PhD in Medicine (Adjunct Professor in the Medical School of the Federal University of Bahia); d PhD in Surgery (Professor of Otorhinolaryngology in the Medical School of the Federal University of Bahia); e PhD in Human Pathology (Coordinator of the Histotechnology Laboratory at the Oswaldo Cruz Insttute Foundaton in Bahia)

**Keywords:** asthma, HTLV-1 infections, rhinitis

## Abstract

The nasal histopathology of HTLV-1 carriers with chronic rhinitis is unknown.

**Objective:**

To describe the histopathological features of HTLV-1 carriers with chronic rhinitis.

**Materials and Methods:**

Biopsies of nasal mucosa of ten HTLV-1 carriers with chronic rhinitis (eight patients with allergic rhinitis and two patients with non-allergic rhinitis) were studied using a light microscope. Samples from ten patients with allergic rhinitis not infected with HTLV-1 were used as controls.

**Results:**

Subepithelial fibrosis was more pronounced in patients with allergic rhinitis infected with HTLV-1 (*p*=0.01), while the basement membrane thickness was greater in controls (*p*=0.03). There was a trend towards less eosinophilia and edema among those infected with HTLV-1, without statistical significance (*p*=0.2). For the lymphocytic infiltrate, there was no difference between infected and not infected patients with allergic rhinitis (*p*=1.0). Subepithelial fibrosis associated to moderate or small number of lymphocytes were found in the two HTLV-1 carriers with non-allergic rhinitis.

**Conclusions:**

This study suggests HTLV-1 may modify the histopathology of allergic rhinitis, especially by promoting subepithelial fibrosis, and may be related to chronic non-allergic rhinitis with lymphocytic infiltrate.

## INTRODUCTION

The Human T-lymphotropic Virus Type 1 (HTLV-1) is a human retrovirus with tropism for T-cells. Diseases such as adult T-cell leukemia and myelopathy are strongly associated with HTLV-1 infection^1^. Other inflammatory conditions including interstitial pneumonia and alveolar/bronchial disorders related to HTLV-1, have been described in case reports^1^, ^2^, ^3^. However, most individuals infected by HTLV-1 are clinically asymptomatic and referred to as carriers.

While HTLV-1 infection is associated to lymphocytic infiltration in the involved tissues, rhinitis allergic – the most common type of chronic rhinitis – and asthma are characterized by eosinophilic infiltration of the nasal and bronchial mucosa respectively^4^, ^5^. Although the histologic characteristics of the nasal mucosa of individuals with allergic rhinitis and concomitant infection by HTLV-1 are unknown, both HTLV-1 infection and respiratory allergic disease may be associated with fibrosis^3^, ^5^.

Some HTLV-1 carriers present respiratory symptoms suggestive of chronic rhinitis, but the characteristics of the nasal mucosa of these patients are unknown. To know them, therefore, is most desirable, even more so in the city of Salvador in the Brazilian Northeast, where HTLV-1 seroprevalence is the highest in the nation: 1.76%^6^. Histologic analysis of these cases may assist in the investigation of the etiology of chronic rhinitis and of the possible influence of HTLV-1 upon allergic rhinitis. Thus, the purpose of this study is to describe histologic findings of the nasal mucosa of HTLV-1 carriers diagnosed with chronic rhinitis, and compare the histology of allergic rhinitis patients to that of subjects not infected by HTLV-1.

## MATERIALS AND METHODS

### Volunteers

This study was approved by our institution's Ethics Committee and given permit number 09/2002; all participants signed an informed consent form.

Eight asymptomatic HTLV-1 carriers diagnosed with untreated perennial allergic rhinitis were recruited at the Multidisciplinary HTLV-1 Ward to undergo nasal biopsy. The diagnosis of perennial allergic rhinitis was produced from the analysis of each patient's history and physical examination, supported by skin tests for local relevant aeroallergens. Additionally, two HTLV-1 carriers with chronic rhinitis of unknown etiology were analyzed; they were negative for local aeroallergens and their prevailing symptoms were nasal obstruction and coryza.

Patients with intestinal parasites, deviated nasal septum, nasal polyps, viral hepatitis, mycobacterium infection, smokers, and subjects taking steroids and/or immunosuppressants were excluded.

Surgical specimens from elective turbinectomy patients with allergic rhinitis and negative serology for HTLV-1 were used as controls. Therefore, this was a sample of convenience. According to the institution's procedures, the patients in this group used nasal topical steroids for three months before surgery and combined oral antihistamines for three weeks. These patients were kept on nasal steroids for ethical reasons, the same reason why patients without nasal complaints did not undergo nasal biopsy.

### Nasal biopsy

The biopsy samples for HTLV-1 carriers with chronic rhinitis were obtained through the Fokkens modified^7^ approach. Local anesthesia was done using a cotton ball soaked in 10% xylocaine and epinephrine (3 drops of 1:1,000 solution) applied on the lower turbinate. Three samples were collected from each patient from the lower tip of the lower turbinate, 2 cm posteriorly to the anterior border, using a 2.5 mm Gerritsma forceps.

### Morphology

Biopsy samples were fixated in 10% buffered formalin and embedded in paraffin. The 5μm slices were dyed in H&E, periodic acid-Schiff (PAS), and Sirius red, and were then observed in the light microscope by an experienced pathologist in a blinded fashion (EAR). Samples were categorized based on the method described by Shioda & Mishima (1966)^8^. Cell count was performed in a semi-quantitative manner by assessing lymphocytes and eosinophils as follows: ±, occasional cells; +, few cells; + +, moderate number of cells; + + +, large number of cells. The criterion to categorize subepithelial fibrosis, edema, and basement membrane thickening is as follows: 0, none; +, mild; + +, moderate; + + +, intense. For purposes of statistical analysis the grades were converted into a numeric scale, in that 0.5 is equal to ±, 1 being +, and so on and so forth. Epithelial lining was categorized as normal, foliated, or affected by squamous metaplasia.

### Immunohistochemistry

Immunohistochemistry testing of inflammatory cells was done in two HTLV-1 carriers whose allergy tests were negative. The paraffin-embedded slices were analyzed using a panel of antibodies and the standard technique using streptavidin-biotin-peroxidase^9^. The following immunohistochemistry markers were used: T-cell markers T CD45RO (UCHL-1), CD4 (OPD4); B-cell marker CD20; lymphoma marker CD30; and proliferation marker Ki-67 (MIB-1).

### Statistical analysis

The Mann-Whitney test was used to compare mean ages, presence of lymphocytes, eosinophils, subepithelial fibrosis, basement membrane thickening, and nasal mucosa edema among HTLV-1 carriers with allergic rhinitis (case group) and patients with allergic rhinitis and negative serology for HTLV-1 (control group). Fisher's test was used to analyze gender differences. For all tests statistical significance was attributed when *p*<0.05.

## RESULTS

Three males and five females with allergic rhinitis and infected by HTLV-1 had ages ranging between 25 and 55 years (mean 44.5). One 55-year-old female patient also had asthma. In the group of allergic rhinitis patients not infected by HTLV-1 (control group), five patients were males and five were females. Their ages ranged between 22 and 56 years (mean 38.9). There was no statistically significant difference for gender and age among the patients with allergic rhinitis either infected or not by HTLV-1.

The two non-allergic rhinitis patients infected by HTLV-1 were males aged 36 and 42 years.

**Figure 1:**
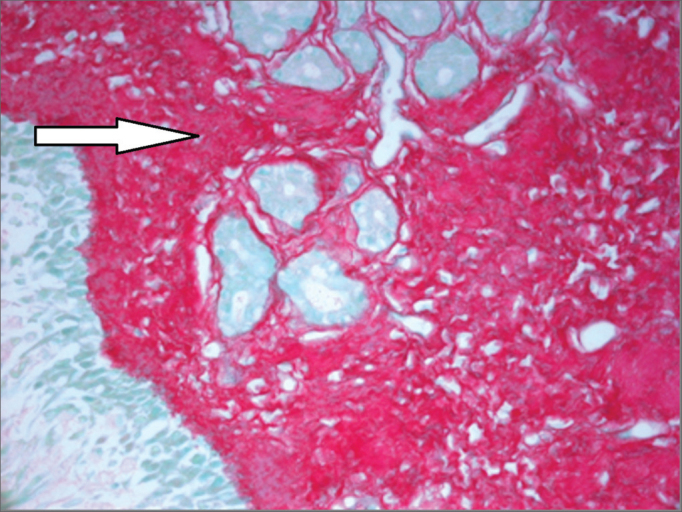
Intense subepithelial fibrosis in nasal mucosa of HTLV-1 carrier with allergic rhinitis. Sirius Red, 400X.

**Figure 2:**
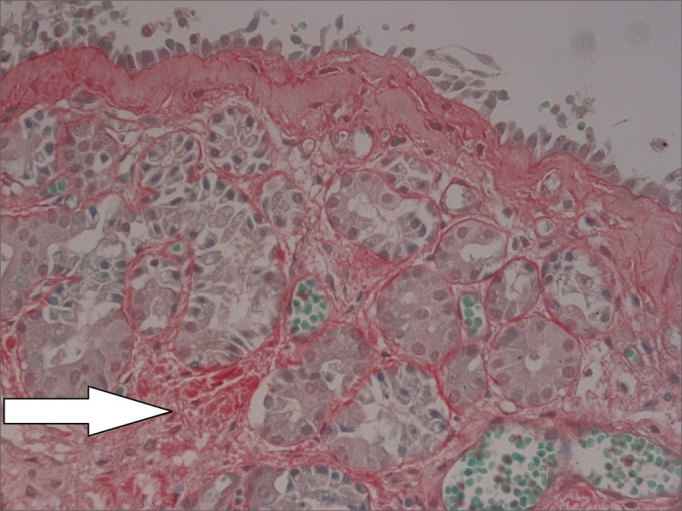
Mild subepithelial fibrosis in nasal mucosa of patient with allergic rhinitis not infected by HTLV-1. Sirius Red, 400X.

Nasal mucosa histology findings can be seen on Tables 1 and 2. The following histological variables presented statistically significant differences: degree of fibrosis, greater on HTLV-1 carriers with allergic rhinitis (*p*=0.01), as seen in the comparison between Figures 1 and 2; and basement membrane thickening, greater in the uninfected control group (*p*=0.03). HTLV-1 carriers tended to have less eosinophilia and edema, but the difference lacked statistical significance (*p*=0.2). There was no difference in terms of lymphocytic infiltrate between infected patients with allergic rhinitis and uninfected subjects (*p*=1.0). Epithelial lining alterations were similar for infected and uninfected patients.

**Table 1:** Semi-quantitative analysis of nasal mucosa histopathology findings of HTLV-1 carriers with allergic rhinitis and non-allergic rhinitis (*).

Patient	Lymphocytic infiltrate	Eosinophilic infiltrate	Subepithelial fibrosis	Edema	Basement membrane thickening	Epithelial lining
1	+	+	++	+	0	Normal
2(*)	++	+	+++	+	+	Normal
3	+	++	+++	+	+	Normal
4	+	+	+++	++	+	Normal
5(*)	+	+	++	0	0	Normal
6	++	+	+	+	0	Foliated
7	+	+	+++	0	0	Squamous metaplasia
8	+	+	+++	+	+	Normal
9	+	+	+++	+	+	Normal
10	+	+	+	0	0	Foliated

(^*^) Patients negative for allergy skin test.

**Table 2:** Semi-quantitative analysis of nasal mucosa histopathology findings of patients with allergic rhinitis and negative serology for HTLV-1 (controls).

Patient	Lymphocytic infiltrate	Eosinophilic infiltrate	Subepithelial fibrosis	Edema	Basement membrane thickening	Epithelial lining
1	+	+++	0	+++	++	Normal
2	++	+++	+	+++	++	Normal
3	+	+	+	+	+++	Normal
4	+	±	++	0	+++	Normal
5	+	++	+	+	+	Normal
6	+	±	+	+	0	Foliated
7	+	±	+	+	0	Foliated
8	+	±	+	++	+	Normal
9	++	+	++	+	++	Squamous Metaplasia
10	+	+++	++	+	++	Normal

**Figure 3:**
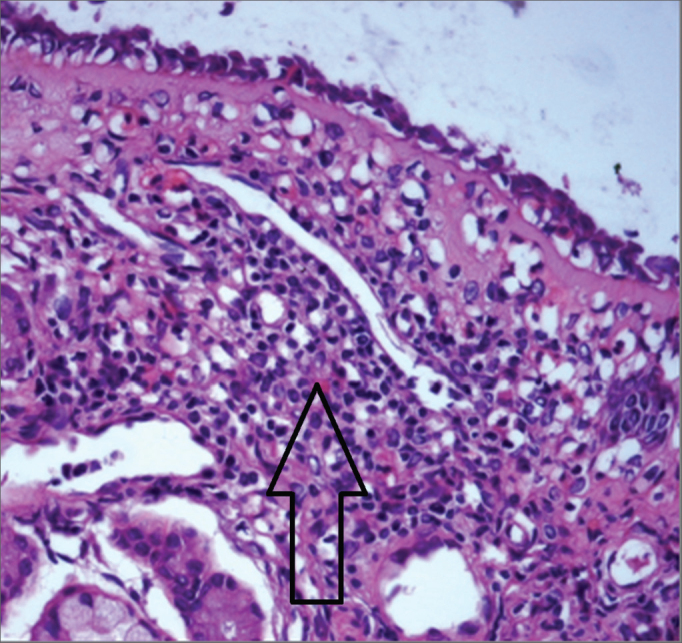
Mononuclear infiltrate in nasal mucosa of HTLV-1 carrier with non-allergic rhinitis. HE, 400X.

Figure 3 shows mononuclear infiltrate with a predominant presence of lymphocytes in HTLV-1 carriers with non-allergic chronic rhinitis. Immunohistochemistry testing of the nasal mucosa of two HTLV-1 carriers with non-allergic rhinitis was positive for T-cell markers CD45RO and CD4, with diffuse and focal distribution respectively. Samples were negative for CD20 (B-cell marker), CD30 (lymphoma marker), and Ki-67 (proliferation marker).

## DISCUSSION

Allergic rhinitis combined with HTLV-1 infection was associated with nasal mucosa subepithelial fibrosis in our study. Basement membrane thickening was observed at a greater degree in allergic rhinitis patients not infected by HTLV-1. Nasal mucosa subepithelial fibrosis was greater in HTLV-1 carriers with allergic rhinitis when compared to uninfected allergic rhinitis patients. The difference in the intensity of subepithelial fibrosis observed between the two groups cannot be explained solely by the antiinflammatory effect of the topical nasal steroids used by the control group, as such association is not established in the literature and the therapy was of short duration. For the patients with allergic rhinitis infected by HTLV-1, one must consider that both allergic rhinitis and HTLV-1 infection may be potentially associated with the fibrosis of the involved tissues. On the other hand, it is not clear why subepithelial basement membrane thickening was lesser in the allergic rhinitis patients not infected by HTLV-1.

Montero et al. (2003) described basement membrane thickening and nasal mucosa subepithelial fibrosis in 92.3% and 92.4%, respectively, of individuals with allergic rhinitis^5^. Chronic inflammation associated with cytokines and mediators such as T-cell growth factor (TGF-β1) and granulocyte–macrophage colony-stimulating factor (GM-CSF) may induce the activation and proliferation of fibroblasts, resulting in the deposition of collagen and other extracellular matrix products^10^. It has been considered that the morphologic remodeling phenomenon in allergic patients occurs less frequently in upper airways than in lower airways, as seen in asthma^10^.

The chronic inflammation associated with HTLV-1 infection causes fibrosis to set in on the involved tissues. Patients with HTLV-1-related myelopathy have early leptomeningeal fibrosis^11^. Airway alveolar fibrosis in HTLV-1 carriers has been documented, but no studies have looked into the nasal mucosa of HTLV-1 carriers^12^.

HTLV-1 infection stimulates the production of GM-CSF, interferon gamma (IFN-γ), interleukin-2 (IL-2), tumor necrosis factor (TNF-α), immune response type 1 cytokines, aside from IL-4, IL-5 and IL-10^13^. On type 2 immune response, seen in allergic inflammation, there is increased production of IL-4, IL-5, and IL-13^14^. Type 1 and type 2 immune response may be mutually impacted both by contra-regulation and synergy^15^. Incidentally, the mutual stimulation between immune responses may explain the significant subepithelial fibrosis seen in patients with allergic rhinitis and HTLV-1 infection, while an inhibitory contra-regulatory effect may be implicated in the cell infiltrate profile seen in the nasal mucosa of these individuals.

Concomitant HTLV-1 infection is associated with the scarce eosinophilia seen in the nasal mucosa of allergic rhinitis patients. This study showed a trend of less nasal eosinophilia in allergic rhinitis patients infected by HTLV-1 when compared to uninfected allergic rhinitis patients treated with steroids. Therefore, this study indicates that HTLV-1 infection may affect nasal eosinophilia to the extent that the findings in this group is, at least, similar to the findings seen in some individuals who were supposedly affected by the therapeutic effect of steroids. Contrary to subepithelial fibrosis, the nasal eosinophilic infiltrate seen in patients with allergic rhinitis may be reduced after only four weeks of treatment with steroids^16^. In the infected group not treated with steroids, inhibitory regulation between the immune profiles of HTLV-1 infection and allergy may be responsible for the discrete eosinophilia seen in the nasal mucosa of these subjects.

Souza-Machado et al. (2005) found prevalence rates of atopy in HTLV-1 carriers of 14.9% against 29.7% in uninfected controls^17^. The diameter of the papules observed in the skin test was smaller in atopic HTLV-1 carriers when compared to atopic uninfected controls^17^. Intercellular Adhesion Molecule 1 (ICAM-1) levels in the nasal fluid of HTLV-1 carriers with allergic rhinitis were lower than those observed on uninfected allergic rhinitis controls^18^. These data indicate that the immune response to HTLV-1 infection may modify allergic manifestations. This histologic study supports these observations and has found incipient eosinophilic infiltrate in HTLV-1 patients with allergic rhinitis.

The two HTLV-1 carriers with allergic rhinitis had mononuclear infiltrate with a predominant presence of T-cells as confirmed by immunohistochemistry. Considering the thesis of the single airway^19^, it is plausible to suppose the existence of rhinitis associated with HTLV-1 analogous to bronchitis and lymphocytic pneumonia as described in HTLV-1 infection^2^, ^20^, ^21^, ^22^, ^23^. Lymphocytic infiltrate has been described in myelitis^11^, uveitis^24^, arthritis^25^, sialadenitis^26^, dermatitis^27^ and nephritis^28^ associated with HTLV-1 infection, but the possible existence of chronic allergic rhinitis not related to HTLV-1 requires further study and more case reports.

## CONCLUSION

This study indicates that HTLV-1 infection may modify the nasal histopathology of allergic rhinitis. This paper indicates that the coexistence of allergic rhinitis and HTLV-1 infection on one same individual may be associated with increased subepithelial fibrosis, less thickening of the subepithelial basement membrane, and a trend towards scarce eosinophilia. Nonetheless, the role of allergy and HTLV-1 infection concomitant to fibrosis on nasal mucosa cell counts require better characterization in future studies. We do not know if fibrosis and nasal lymphocytic infiltrate seen in HTLV-1 carriers are a new entity within the realm of rhinitis.
